# Elaboration of a multimodal MRI-based radiomics signature for the preoperative prediction of the histological subtype in patients with non-small-cell lung cancer

**DOI:** 10.1186/s12938-019-0744-0

**Published:** 2020-01-21

**Authors:** Xing Tang, Xiaopan Xu, Zhiping Han, Guoyan Bai, Hong Wang, Yang Liu, Peng Du, Zhengrong Liang, Jian Zhang, Hongbing Lu, Hong Yin

**Affiliations:** 1 Department of Radiology, Xijing Hospital, Fourth Military Medical University, No. 169 Changle West Road, Xi’an, 710032 Shaanxi People’s Republic of China; 2 School of Biomedical Engineering, Fourth Military Medical University, No. 169 Changle West Road, Xi’an, 710032 Shaanxi People’s Republic of China; 3 Department of Respiratory Medicine, Xijing Hospital, Fourth Military Medical University, No. 169 Changle West Road, Xi’an, 710032 Shaanxi People’s Republic of China; 4 Department of Clinical Laboratory, Shaanxi Provincial People’s Hospital, Xi’an, Shaanxi People’s Republic of China; 5 Departments of Radiology, School of Computer Science and Biomedical Engineering, State University of New York, Stony Brook, NY USA

**Keywords:** Non-small-cell lung cancer, Lung squamous cell carcinoma, Lung adenocarcinoma, Multimodal MRI radiomics features, Clinical features, Nomogram

## Abstract

**Background:**

Non-invasive discrimination between lung squamous cell carcinoma (LUSC) and lung adenocarcinoma (LUAD) subtypes of non-small-cell lung cancer (NSCLC) could be very beneficial to the patients unfit for the invasive diagnostic procedures. The aim of this study was to investigate the feasibility of utilizing the multimodal magnetic resonance imaging (MRI) radiomics and clinical features in classifying NSCLC. This retrospective study involved 148 eligible patients with postoperative pathologically confirmed NSCLC. The study was conducted in three steps: (1) feature extraction was performed using the online freely available package with the multimodal MRI data; (2) feature selection was performed using the Student’s *t* test and support vector machine (SVM)-based recursive feature elimination method with the training cohort (*n* = 100), and the performance of these selected features was evaluated using both the training and the validation cohorts (*n* = 48) with a non-linear SVM classifier; (3) a ***Radscore*** model was then generated using logistic regression algorithm; (4) Integrating the ***Radscore*** with the semantic clinical features, a radiomics–clinical nomogram was developed, and its overall performance was evaluated with both cohorts.

**Results:**

Thirteen optimal features achieved favorable discrimination performance with both cohorts, with area under the curve (AUC) of 0.819 and 0.824, respectively. The radiomics–clinical nomogram integrating the ***Radscore*** with the independent clinical predictors exhibited more favorable discriminative power, with AUC improved to 0.901 and 0.872 in both cohorts, respectively. The Hosmer–Lemeshow test and decision curve analysis results furtherly showed good predictive precision and clinical usefulness of the nomogram.

**Conclusion:**

Non-invasive histological subtype stratification of NSCLC can be done favorably using multimodal MRI radiomics features. Integrating the radiomics features with the clinical features could further improve the performance of the histological subtype stratification in patients with NSCLC.

## Background

Lung cancer is the most common diagnosed cancer and the leading cause of cancer death for both men and women [1–5]. As the most common type of lung cancer, non-small-cell lung cancer (NSCLC) comprises 85% of the primary lung malignancies, and the 5-year survival rate is less than 20% [1–3, 5, 6]. According to the *New England Journal of Medicine* [5], non-small-cell lung cancer (NSCLC) can be divided into three major histologic subtypes, namely squamous cell carcinoma (LUSC), adenocarcinoma (LUAD), and large-cell lung cancer, and all these subtypes are malignant tumors, among which LUSC and LUAD constitute approximately 35% and 60% of the primary NSCLC cases, respectively [1, 2, 4, 5, 7]. LUSC and LUAD have their own tissue characteristics, anatomical site and location, and glucose metabolism, which indicates different optimal treatment decisions to improve the clinical outcomes [4–7]. Therefore, it is very crucial to accurately confirm the histological subtype of the NSCLC prior to the treatment decisions [6].

Clinically, the histopathological analysis of the tumor tissues by biopsy is the first-line reference in identifying the NSCLC subtypes [4–8]. It is an invasive diagnostic process and full of risk in actual practices [6]. Besides, considering the spatial and temporal heterogeneity of the tumors, the biopsy can only extract very limited portions of the target tissue, incapable of a complete characterization of tumor properties [1, 7]. Hence, a non-invasive approach for the preoperative, accurate identification of LUSC and LUAD with the whole tumor site is required.

In recent years, computed tomography (CT) and magnetic resonance imaging (MRI) have been widely used for preoperative detection and diagnosis of lung cancer [1, 7, 9–12]. Compared with the CT, MRI has excellent soft tissue contrast and does not involve the use of ionizing radiation. However, as for the discrimination between LUSC and LUAD, it is a real challenge for the radiologists to make a visual judgment based on the MRI data. Besides, the performance and consistency of the previous studies varied dramatically [2, 13, 14]. Deep extraction of the quantitative features beneath the MRI data, i.e., radiomics [15–19], for the objective accurate discrimination between LUSC and LUAD deserves more attention.

Currently, the radiomics strategies based on multimodal MRI data, including the T2-weighted images (T2WI), diffusion-weighted images (DWI) and the corresponding apparent diffusion coefficient (ADC) images, have been widely used for breast cancer, bladder cancer, nasopharyngeal carcinoma and glioblastoma subtypes discrimination and outcomes prediction [15–26]. Whether the radiomics features extracted from multimodal MRI could reflect the significant differences of tissue distribution patterns between LUSC and LUAD, remains inconclusive up to now.

Therefore, ***the first aim*** of this study was to investigate whether the radiomics features extracted from multimodal MRI could significantly reflect the tissue distribution differences between LUSC and LUAD, and explore a feasible way for preoperative discrimination of LUSC and LUAD. To achieve this goal, five feature categories were employed in this study, including the histogram features, the Haralick features of co-occurrence matrices (CM features hereafter) [27], and features derived from the run length matrix (RLM features hereafter) [28], the neighborhood gray-tone difference matrix (NGTDM features hereafter) [29], and the gray-level size zone matrix (GLSZM features hereafter) [30], to fully characterize the global, local and regional heterogeneity differences of tumor tissues between LUSC and LUAD [19].

Considering that the semantic clinical features like age, sex, smoking history, size, location, the longest diameter (LD) and its longest perpendicular diameter (LPD) of the target lesion, and carcinoembryonic antigen (CEA) are closely related to lung cancer [4, 5], ***the second aim*** of this was to investigate whether integrating the radiomics features with these clinical features could further improve the diagnostic performance.

## Results

### Clinical characteristics of the patients

The baseline demographics and clinical information of the patients in both the training and the validation cohorts were collected from the archival medical documents, as shown in Table 1. The statistical analyses showed no significant differences between the training and validation cohorts in term of all these factors.

**Table 1:** Baseline demographics of the patients involved in this research

Characteristics	Training cohort (*n* = 100)	Validation cohort (*n* = 48)	*p* value
Age, years			0.055
Median [range]	58 [20, 76]	61 [42, 83]	
Sex, no. (%)			0.220
Male	79/100 (79%)	33/48 (68.75%)	
Female	21/100 (21%)	15/48 (31.25%)	
Smoking, no. (%)			0.707
Yes	70/100 (70%)	32/48 (66.7%)	
No	30/100 (30%)	16/48 (33.3%)	
Side, no. (%)			0.389
Upper left lobe	34/100 (34%)	12/48 (25%)	
Lower left lobe	14/100 (14%)	8/48 (16.7%)	
Upper right lobe	22/100 (22%)	12/48 (25%)	
Middle right lobe	4/100 (4%)	2/48 (4.2%)	
Lower right lobe	26/100 (26%)	14/48 (29.1%)	
Location, no. (%)			0.216
Peripheral	63/100 (63%)	25/48 (52.1%)	
Central	37/100 (37%)	23/48 (47.9%)	
LD, mm^a^			0.230
Median [range]	54 [10, 115]	43.5 [15, 100]	
LPD, mm^a^			0.838
Median [range]	36.5 [8, 77]	35 [11, 90]	
CEA, (ng/ml)^a^			0.380
Median [range]	4.7 [0.486, 1135]	7.11 [1.19, 646.4]	
Histological subtype, no. (%)			0.164
Squamous cell carcinoma	50/100 (50%)	18/48 (37.5%)	
Adenocarcinoma	50/100 (50%)	30/48 (62.5%)	

^a^LD, LPD and CEA indicate the longest diameter, the longest perpendicular diameter, and carcinoembryonic antigen, respectively

### Performance of the optimal features selected for the discrimination between LUSC and LUAD

After Student’s *t* tests for all the 1404 radiomics features in the training cohort, 534 features showed significant differences between LUSC and LUAD, indicating that the multimodal MRI radiomics features describing the tissue distribution patterns, could well reflect the tissue distribution differences between LUSC and LUAD.

**Fig. 1:**
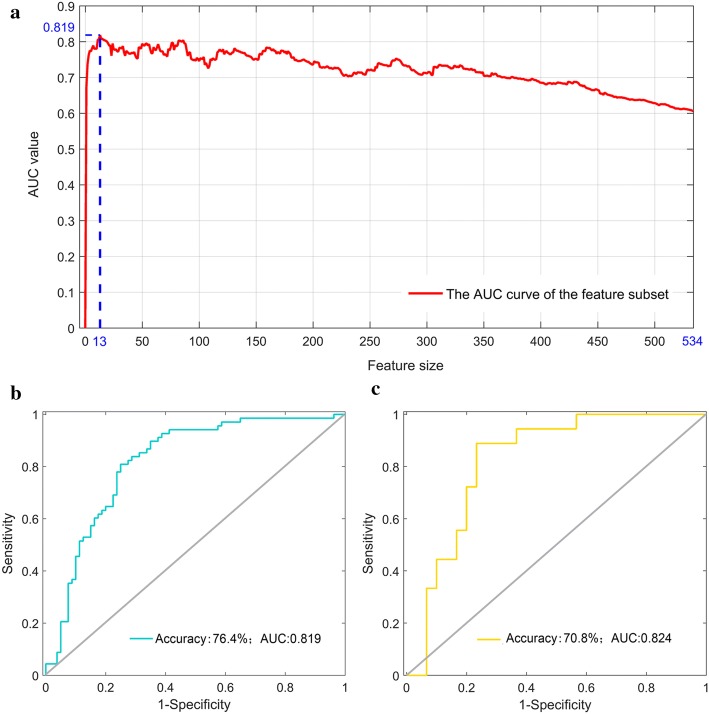
Optimal features selection process and their classification performance with both cohorts: **a** features selection process (AUC indicates the area under the curve of the receiver operating characteristic); **b** the performance of the selected features in the training cohort; **c** the performance of the features with the validation cohort

With a non-linear support vector machine (SVM)-based recursive feature elimination (SVM-RFE) approach further applied in these significant features, 13 features were finally selected as the optimal features, as shown in Fig. 1a. The discrimination performance of the optimal features in both the training and validation cohorts was then evaluated using a radial basis function-based non-linear SVM classifier with the LUSC patients labeled as “1” and the LUAD labeled as “−1”, as shown in Fig. 1b, c, indicating a favorable prediction performance.

### The *Radscore* calculation

**Fig. 2:**
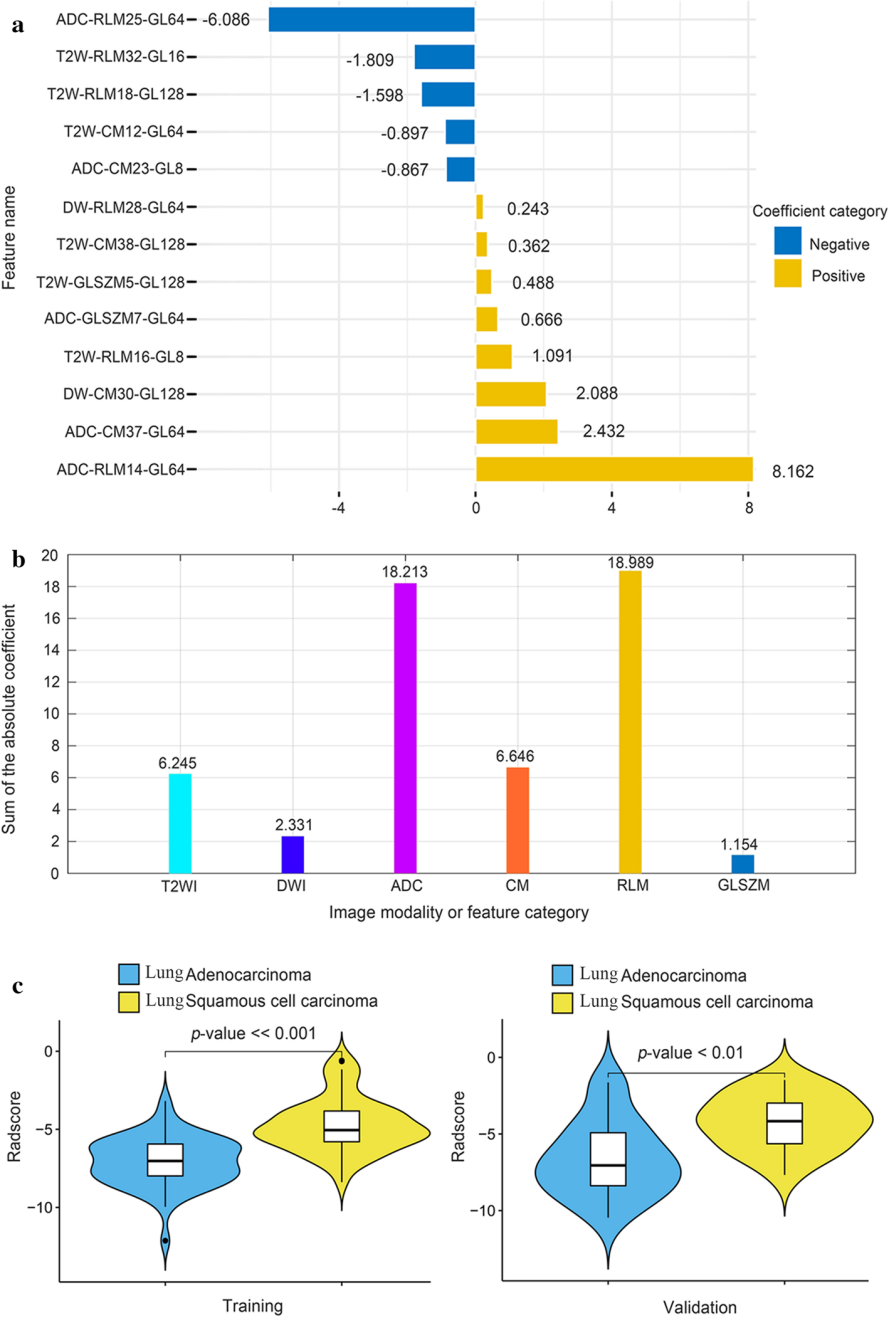
***Radscore*** generation and its inter-group distribution (ADC, DWI, T2WI, CM, RLM, GLSZM and GL represent the apparent diffusion coefficient, the diffusion-weighted images, the T2-weighted images, the co-occurrence matrices, the run length matrix, the gray-level size zone matrix, the gray level, respectively): **a** coefficient map of the 13 features; **b** sum absolute coefficients of the features with different modalities or categories; **c** the distribution and inter-group analyses of the ***Radscore***

To simplify the prediction model furtherly, a ***Radscore*** formula based on these optimal features was generated by using a logistic regression algorithm, and the coefficient for each feature is listed in Fig. 2a. The intercept of the formula was 2.975. Figure 2b shows the sum of the absolute coefficients of these features in terms of image modalities or feature categories, from which we noticed that (1) the RLM feature category had the highest weight in the ***Radscore*** formula, and (2) the features derived from ADC maps contributed most in the ***Radscore*** calculation. Using the formula, the ***Radscore*** of each patient in both cohorts was calculated, which exhibited significant differences between the LUSC and LUAD patients (*p* value < 0.01), as shown in Fig. 2c.

### Performance of the radiomics–clinical nomogram in the discrimination task

In order to further improve the discrimination performance, the clinical features and the ***Radscore*** were jointly considered. After the univariate and multivariable analyses of the ***Radscore*** and the clinical features, ***age, smoking, location, LD, LPD***, and ***Radscore*** were identified as independent predictors for the discrimination task, as shown in Table 2.

**Table 2:** Univariate and multivariable regression analyses of the *Radscore* with primary clinical features for the histological subtype prediction of NSCLC in the training cohort

Indicators	Univariate analysis				Multivariable analysis			
	OR^a^	95% CI		*p* value	OR	95% CI		*p* value
		Lower	Upper			Lower	Upper	
Age	2.481	1.368	4.499	< 0.05	*3.994*	*1.421*	*11.228*	*< 0.05*
Sex	0.068	0.015	0.313	≪ 0.05	0.413	0.015	10.889	0.59
Smoking	0.148	0.053	0.409	≪ 0.05	*0.104*	*0.012*	*0.947*	*< 0.05*
Side	0.353	0.127	0.981	0.051	–	–	–	–
Location	2.191	0.954	5.028	0.06	–	–	–	–
LD^a^	1.991	1.061	3.734	< 0.05	*0.048*	*0.005*	*0.511*	*< 0.05*
LPD^a^	3.246	1.651	6.379	≪ 0.05	*9.807*	*1.420*	*67.719*	*< 0.05*
CEA^a^	0.784	0.635	0.968	< 0.05	0.661	0.488	1.129	0.062
*Radscore*	11.128	4.100	30.198	≪ 0.01	*74.937*	*9.339*	*601.32*	*≪ 0.01*

The underlined values indicate statistical significance with *p* value < 0.05 after the univariate analysis

The italics underlined values indicate statistical significance with *p* value < 0.05 after the multivariable analysis

^a^LD, LPD, CEA and OR indicate the longest diameter, the longest perpendicular diameter, carcinoembryonic antigen, and odds ratio, respectively

**Fig. 3:**
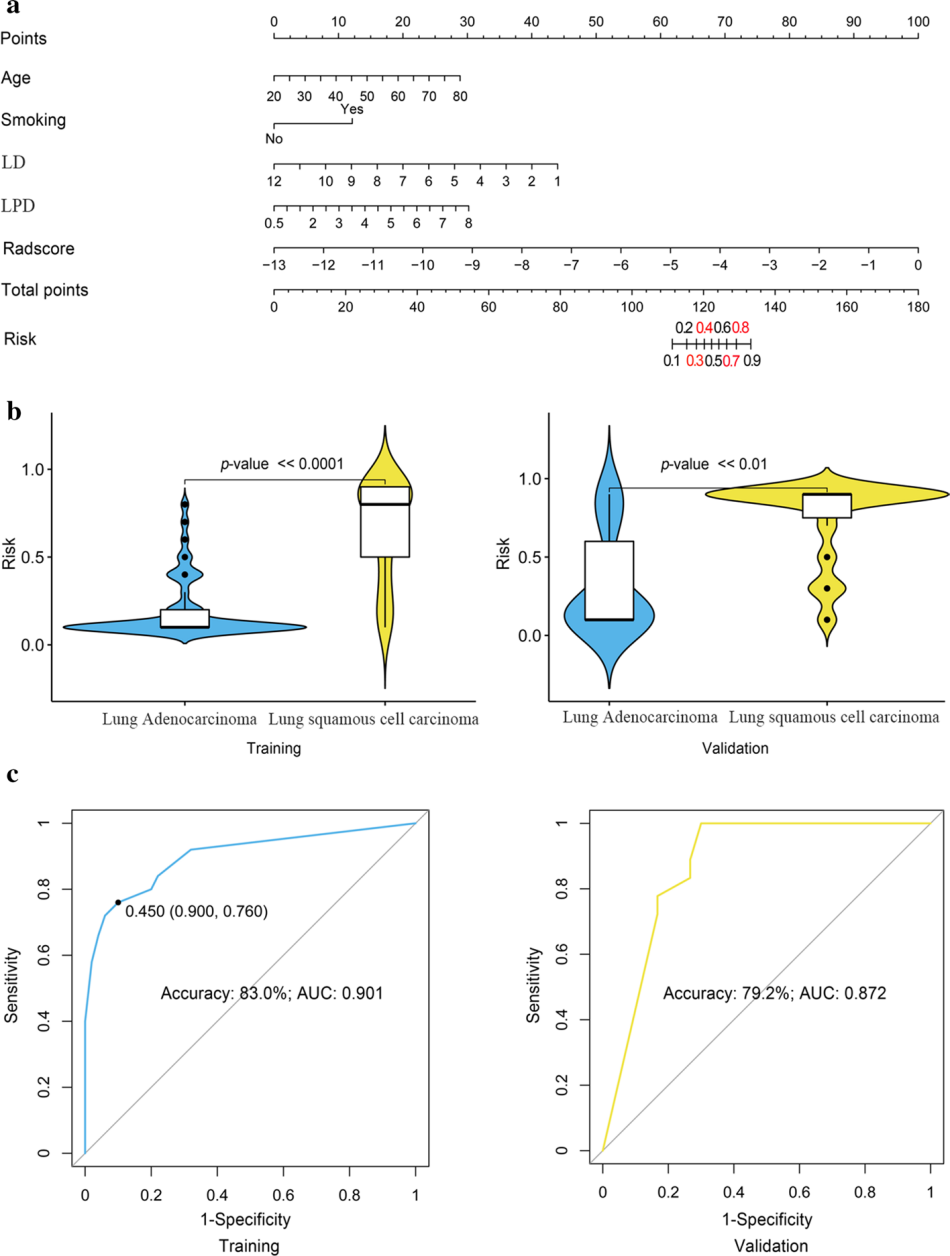
Construction and validation of the nomogram: **a** development of the nomogram based on the ***Radscore*** and independent clinical predictors (LD and LPD represent the longest diameter and the longest perpendicular diameter, respectively); **b** the risk calculated and its statistical inter-group distribution differences; **c** performance verification (AUC indicates the area under the curve of the receiver operating characteristic)

Then, the radiomics–clinical nomogram was developed by integrating these five predictors, as shown in Fig. 3a. Based on the nomogram, the risk of each patient for being identified with LUSC was quantitatively calculated. Figure 3b shows the significant differences of the risk distribution between the LUSC and LUAD patients in both cohorts (*p* value  ≪ 0.01). With this nomogram, the discriminative performance was greatly improved, as shown in Fig. 3c and Table 3. With a risk threshold of 0.450, the prediction accuracy and AUC were improved to 83.0% and 0.901 in the training cohort and 79.2% and 0.872 in the validation cohort, respectively. Besides, comparing the performance of proposed approach (Fig. 3c) with the existing techniques on the basis of *t* test + SVM (Fig. 1b), the former achieved a much higher predictive precision in terms of the accuracy and AUC.

**Table 3:** Performance of the radiomics–clinical nomogram in discriminating between lung squamous cell carcinoma (LUSC) and lung adenocarcinoma (LUAD) in both training and validation cohorts

Cohort	Sen^a^	Spe^a^	Acc^a^	AUC^a^	95% CI		*p* value
					Lower	Upper	
Training	90.0%	76.0%	83.0%	0.901	0.842	0.960	< 0.05
Validation	88.9%	73.3%	79.2%	0.872	0.779	0.965	< 0.05

^a^Sen, Spe, Acc and AUC indicate the sensitivity, specificity, accuracy and area under the curve of the receiver operating characteristic curve, respectively

**Fig. 4:**
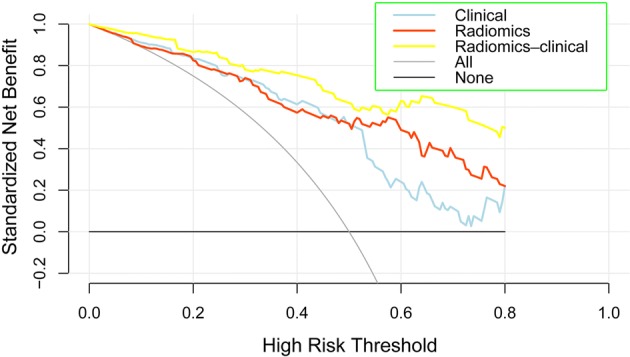
Clinical usefulness assessed by using the decision curve analysis indicating a greater net benefit than individually using the clinical model or the radiomics model

Additionally, the Hosmer–Lemeshow test yielded a *p* value of 0.893 without statistical significance, suggesting a favorable agreement between the predicted and observed results using this nomogram model. Clinical usefulness was assessed by decision curve analysis, as shown in Fig. 4, which indicated a greater net benefit than individually using the clinical model or the radiomics model as the risk larger than 0.1.

## Discussion

In this study, we developed and validated a radiomics–clinical nomogram incorporating the multimodal MRI-based radiomics features and the primary clinical features for the preoperatively individualized discrimination and the risk stratification of the patients with LUSC and LUAD. The results of using the nomogram in both the training and the validation cohorts demonstrate a favorable discriminative power and clinical usefulness, suggesting that the proposed nomogram could be an effective, non-invasive and absolutely safe manner for the preoperative identification of histological subtypes of NSCLC.

In recent years, the MRI was widely used for a variety of cancers diagnosis like glioblastoma, nasopharyngeal carcinoma, lung cancer, bladder cancer, and prostate cancer [13, 17, 31–38]. Most of the diagnoses were based on the visual interpretation of the experts. With the rapid development of multimodal MRI and image analysis techniques, radiomics approaches based on multimodal MRI data have recently drawn great attention for cancer properties and subtype prediction and prognosis, preoperatively [15–22]. However, as for the NSCLC histological subtype discrimination, the feasibility and performance of the multimodal MRI-based radiomics approach remain largely unknown up to now. Therefore, we aimed to (i) investigate whether the radiomics features extracted from multimodal MRI could significantly reflect the tissue distribution differences between LUSC and LUAD, exploring a feasible way for preoperative discrimination between LUSC and LUAD and (ii) verify if integrating the radiomics features with the clinical features would further improve the discriminative power in this study.

Due to the different original grayscales of the T2WI, DWI and ADC images, grayscale standardization was indispensable prior to the CM, RLM, NGTDM and GLSZM features calculation in the process of radiomics feature extraction. In this study we implemented a multi-grayscale normalization strategy with five commonly normalized grayscales based on the previous researches [18, 19], to extract more features potentially useful for the discrimination task. With the Student’s *t* test and SVM-RFE approaches jointly used for feature selection, 13 features with significant inter-group differences were determined as the optimal features, and their classification results with both the training and validation cohorts demonstrated the feasibility and fairly good performance of the multimodal MRI-based radiomics strategy for the preoperative discrimination of patients with LUSC or LUAD.

Although the SVM classifier has several drawbacks, including the apparent complexity increase and large time consumption for large database [39, 40], its merits are also very apparent. Specifically, as for the small samples like the circumstance in this study, SVM can usually get favorable results using the limited datasets in the training set [39–41]. Besides, the generalizability of the SVM classifier is also remarkable in terms of the small and limited datasets [39, 40].

Among these optimal features selected, the sum of the absolute coefficients of the RLM features was the highest, potentially demonstrating that features well reflecting the regional heterogeneity of tumor tissues could better characterize the heterogeneous differences between LUSC and LUAD. In addition, the sum of the absolute coefficients of the features extracted from ADC maps was exceedingly the highest, indicating that the ADC maps could well reflect the histological differences between LUSC and LUAD of NSCLC.

Concerning that the primary clinical features like age, sex, smoking, side, location, LD and LPD of the target lesion, and CEA are commonly used for the clinical diagnosis of patients with lung cancer, whether incorporating these factors with the ***Radscore*** generated by the 13 optimal features would improve the discriminative performance was in great need to answer. The univariate and multivariable analyses results showed that age, smoking, the longest diameter of the target lesion, the longest perpendicular diameter of the target lesion and the ***Radscore*** were independent predictors for the discrimination task. Based on these predictors, a nomogram was then generated. The discriminative performance of the nomogram was evidently better than that of the radiomics model, apparently demonstrating that integrating the radiomics features with the primary clinical features could further improve the discriminative power. Besides, the Hosmer–Lemeshow test and the decision curve analysis results further demonstrate good predictive precision and clinical usefulness of the nomogram.

In recent years, only a few CT-based studies have investigated the performance of the radiomics strategy for preoperatively differentiating LUSC from LUAD. Previously, Zhu et al. [7] used 485 radiomics features extracted from 81 patients’ CT images to generate a radiomics signature for the discrimination task. It finally achieved an AUC of 0.893 in the validation cohort (48 patients) [7]. In another study, Linning et al. [42] adopted the preoperative non-enhanced CT images and dual-phase chest contrast-enhanced CT images acquired from 90 LUAD and 84 LUSC patients with the radiomics strategy to generate two predictive models, respectively. And the AUC of these models were 0.801 and 0.806, respectively. In a more recent study [2], Bashir et al. employed 115 radiomics features extracted from 106 patient’s CT images with the random forest classifier to develop the predictive model. And its performance in the validation cohort (100 patients) was really poor, with AUC of only 0.56 [2]. Comparing with the results of these studies, the proposed algorithm in our study achieved a favorable and compatible performance, with AUC of 0.901 and 0.872 in the training cohort and the validation cohort, respectively. Besides, the proposed approach in our study could also realize the quantitative estimation and risk stratification for patients with LUSC and LUAD, promisingly working as an effective and complementary tool to help the clinicians make appropriate treatment decisions.

Apart from the current study, development of the quantitative image-based diagnostic models for disease definition has received unprecedented attention these years [2, 7, 18, 25, 38, 43–47], not only in the field of cancer diseases, but also in more broad research fields, such as retinal diseases [43, 44], diabetes [46], calcaneal fracture [45], and mental disorders [36]. These models have achieved favorable performance in diseases diagnoses and understanding, demonstrating the great power and promising application of these approaches for clinical practice. However, the results of this study should be carefully interpreted due to several limitations. First, inherent bias might exist because of the retrospective nature of this current study with relatively limited patient cohorts. A larger amount of participants from two or more clinical centers are needed to further validate the overall performance of the proposed approach. Moreover, other potentially clinical factors, such as gene mutations and key molecular biomarkers, are not included in the current study because of the incomplete data in the archival database, and should be further analyzed.

## Conclusions

The proposed multimodal MRI-based radiomics signature could be an effective tool for the quantitative description and discrimination of NSCLC subtypes. Additional integration of the significant clinical factor with the signature further improves the discriminatory power. Extensive multicenter validations of the proposed approach are required prior to real clinical application.

## Methods

**Fig. 5:**
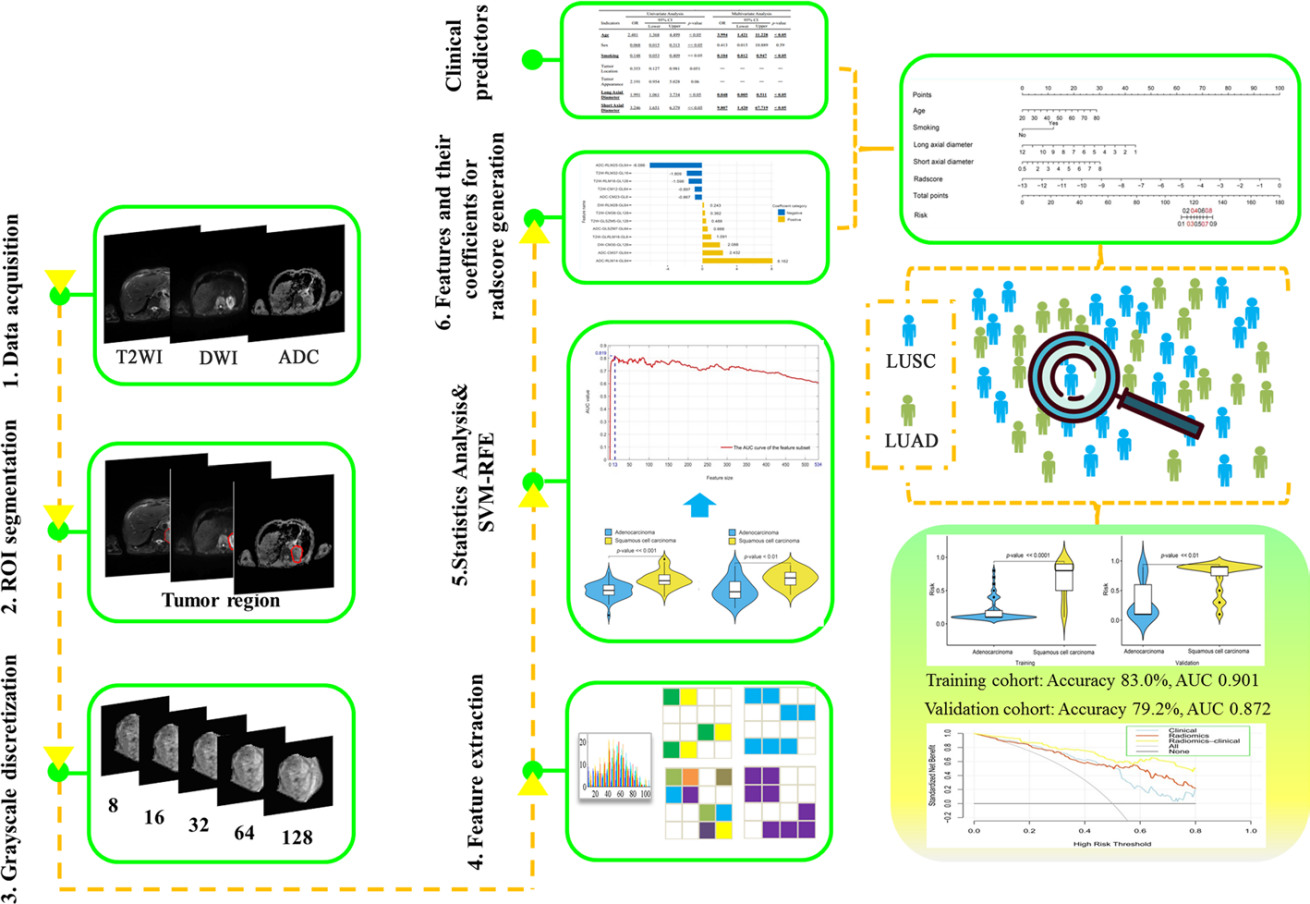
The overall schematic outline of this study for the preoperative discrimination between squamous cell carcinoma (LUSC) and adenocarcinoma (LUAD)

The institutional ethics review board of the Xijing hospital approved this retrospective study and waived the requirement for informed content. Overall methodology of this study is shown in Fig. 5.

### Patients

**Fig. 6:**
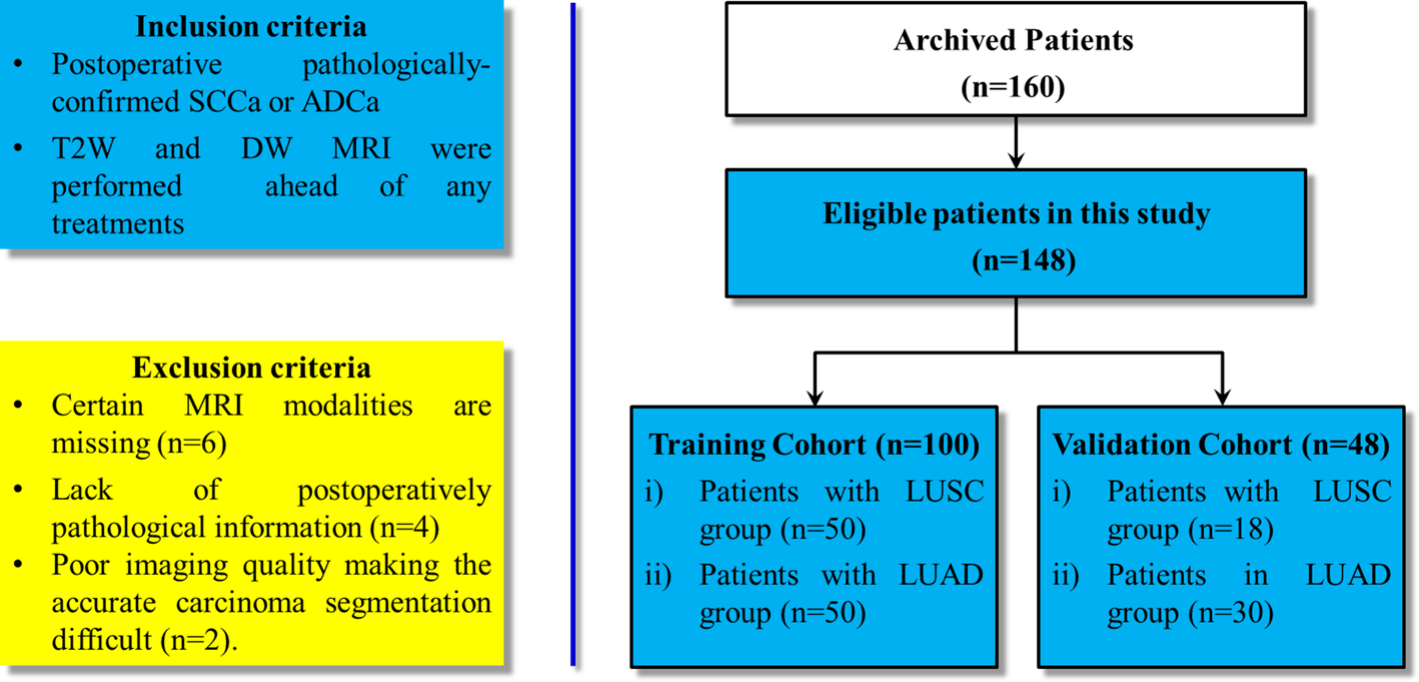
Inclusion and exclusion criteria of this study (LUSC and LUAD represent the lung squamous cell carcinoma and lung adenocarcinoma, respectively)

This study consisted of 148 eligible patients in which all the lesions we included were postoperatively confirmed with LUSC or LUAD from a single clinical center between January 2015 and December 2018. Then, their preoperative imaging datasets were enrolled and used for model development. If he/she is a healthy subject, the tumor mass will not be observable and delineated for feature extraction. Therefore, it is impossible and unnecessary to launch the model for the prediction. According to the previous studies [21, 37, 48–50], we randomly allocated the entire datasets into the training cohort and the validation cohort. Therefore, 100 patients (79 males and 21 females) with postoperative pathologically confirmed LUSC (*n* = 50) or LUAD (*n* = 50) were allocated as the training cohort for model development, and 48 patients (33 males and 15 females) were allocated as the independent validation cohort. The overall inclusion and exclusion criteria are illustrated in Fig. 6. Only the lesions greater than 8 mm were included in this study to ensure sufficient counting statistics and consistent region of interest analysis. Besides, the primary clinical features including age, sex, smoking, side, location, LD, LPD, and CEA were obtained from the archival medical records. Postoperative histological subtypes were used as the true label of the NSCLC patients.

### Image acquisition and region of interest delineation

All patients underwent MRI using a 1.5 T scanner (MAGNETOM Aera, Siemens Medical Solutions, Erlangen, Germany) with an 8-channel phased-array torso coil. MRI sequences, including T2-weighted and Diffusion-weighted MRI sequences, were performed to obtain the corresponding images. The ADC maps were derived automatically from the DWI using a biexponential model with b values of 50 and 800 s/mm^2^. The primary parameters of these sequences were described in the Additional file 1.

**Fig. 7:**
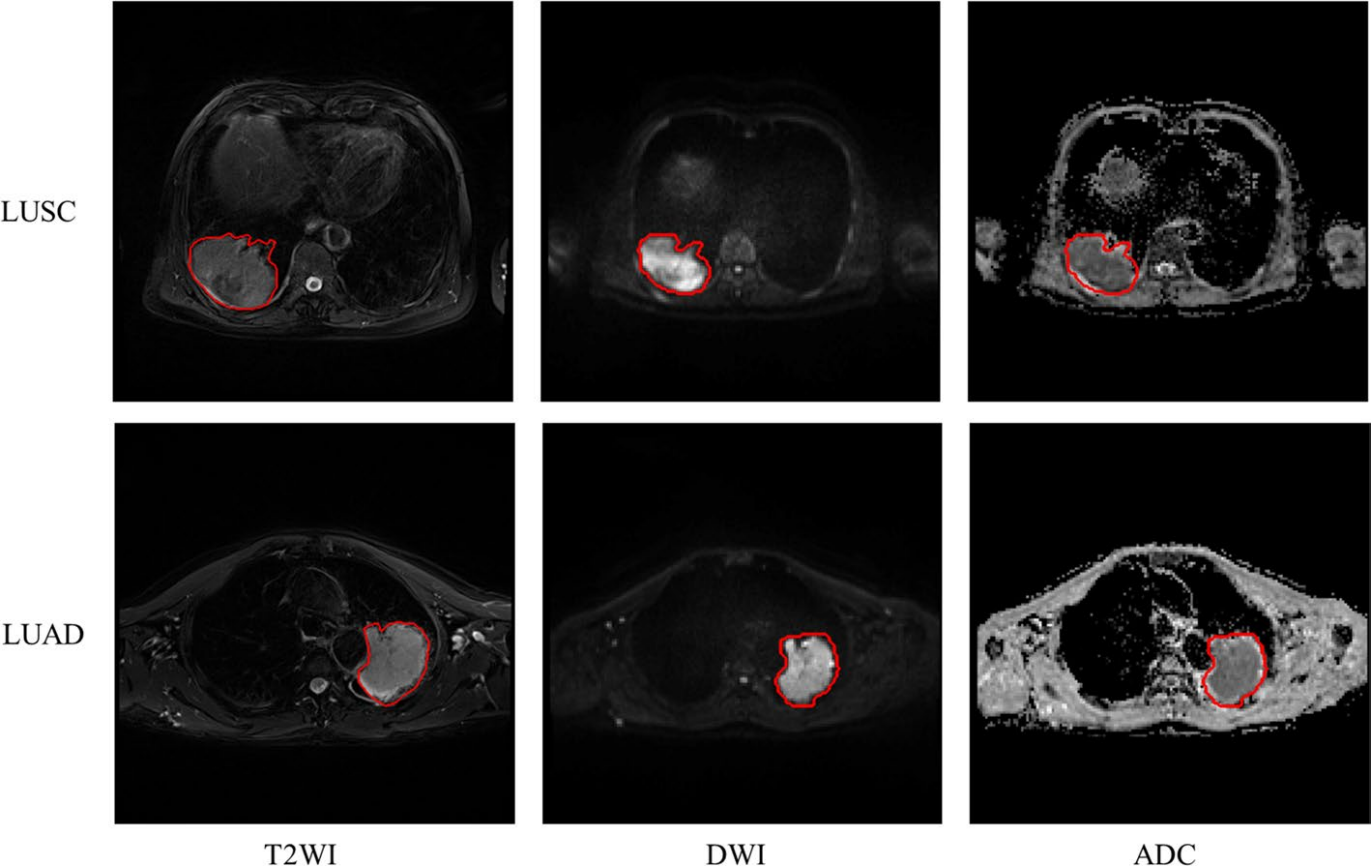
Examples of the delineated lung squamous cell carcinoma (LUSC) and lung adenocarcinoma (LUAD) on the multimodal MRI data

Before tumor region of interest (ROI) delineation, the axial image slice for each MRI modality was selected based on obtaining the largest area of the archived tumor with the maximal size in each patient’s lung region. Then, a manually depicted polygonal ROI was used to segment the tumor area on the selected images. Two radiologists with 9 and 5 years of MRI interpretation experience of lung cancer, independently performed tumor delineation with a custom-developed package. Then, divergence of their delineation results was carefully corrected by consensus. Considering that the ADC maps were calculated from the DWI using the biexponential model, the tumor ROIs obtained from the DWI were mapped onto the ADC maps to extract the corresponding tumor regions. Examples of the ROI delineation results are illustrated in Fig. 7.

### Feature extraction

The image features including 8 histogram features, 39 CM features, 33 RLM features, five NGTDM features [29], and 15 GLSZM features [30], were extracted from the tumor ROIs of the MRI data to fully characterize the local, regional and global tissue distribution variations of the tumor [18, 50]. Detailed feature information is shown in Additional file 1: Table S1. Due to the different grayscales of the original T2W, DW and ADC images, which is prior to the second-order (CM features) and higher-order (RLM, NGTDM, and GLSZM features) texture feature extraction, a multi-grayscale standardization strategy was performed on all the tumor ROIs delineated from three MRI modalities by using 8, 16, 32, 64, and 128 grayscales [15, 17, 22]. Then, a total of 1404 features were obtained, and their values were linearly normalized in the range of − 1 to 1 to reduce the computational burdens. The feature extraction process was performed using a publicly shared MATLAB package available online [18, 19, 51].

### Feature selection, predictive performance evaluation, and Radscore generation

Multiple test methods were utilized in combination to select the optimal features for the discrimination between LUSC and LUAD of patients with NSCLC. First, the Student’s *t* test was employed to select the features with statistically significant differences between the two groups in the training cohort. Subsequently, SVM-RFE approach was adopted to select an optimal feature subset from these features in the training cohort [37], and its differentiation performance was evaluated with both the training and the validation cohorts. Detailed description on SVM-RFE has been summarized in the Ref. [18, 19]. After that, a logistic regression algorithm was performed with these optimal features in the training group to obtain the coefficient of each feature and the intercept for ***Radscore*** formula generation [18, 19, 37]. Based on the formula, the ***Radscore*** of each patient in the two patient cohorts was then computed for the further analysis [18, 19, 37].

### Radiomics–clinical nomogram development and its predictive performance assessment

After exploring the feasibility and evaluating the performance of the radiomics model for the discrimination between LUSC and LUAD, whether the inclusion of both radiomics and the primary clinical features could improve the diagnostic accuracy for the discrimination task was further investigated. First, the univariate and multivariable regression analyses were performed with the ***Radscore*** and the clinical features in the training cohort to determine the independent predictors for the discrimination between LUSC and LUAD [19, 49, 52]. Then, the nomogram based on these independent predictors was developed using the training cohort [19, 49, 52], and its predictive performance was quantitatively assessed in terms of the sensitivity, specificity, accuracy, and AUC of receiver operating characteristic (ROC) using both the training and validation cohorts [19, 49, 52]. Among these metrics, sensitivity measures the percentage of positives samples which are correctly identified, specificity evaluates the proportion of negatives samples which are correctly predicted, and the accuracy is the ratio of all samples which are correctly identified, as shown in Eq. 1, where TP, TN, FP, FN are the abbreviations of true positive, true negative, false positive and false negative, respectively [52–54]. The AUC measures the area under the curve of the receiver operating characteristic (ROC) after the test, assessing the general performance of the predictive model [53–55]. The Hosmer–Lemeshow test and decision curve analysis were performed to verify the precision and net benefit of the nomogram in clinical applications [56].

1 $$ \left\{ \begin{aligned} {\text{Sensitivity}} = \frac{\text{TP}}{{{\text{TP}} + {\text{FN}}}} \hfill \\ {\text{Specificity}} = \frac{\text{TN}}{{{\text{TN}} + {\text{FP}}}} \hfill \\ {\text{Accuracy}} = \frac{{{\text{TP}} + {\text{TN}}}}{{\begin{array}{*{20}c} {{\text{All}}\,{\text{samples}}} \\ \end{array} }} \hfill \\ \end{aligned} \right. $$

### Statistical analysis

All statistical analyses were performed using R statistical software (version 3.4.4., × 64), R Foundation for Statistical Computing, Vienna, Austria. URL http://www.R-project.org/ and two-sided *p* values less than 0.05 were considered to be significant [19, 49, 52]. Univariate and multivariable regression analyses were applied to identify independent predictors for the discrimination task [19, 49, 52]. The Hosmer–Lemeshow test was performed to quantitatively assess the calibration and agreement between the predicted and observed results, and decision curve analysis was employed to evaluate the clinical usefulness of the proposed nomogram model.

### Data statement

The datasets in this study are currently not available for freely public access owing to the patient privacy concerns, but may be obtainable from the corresponding authors with the reasonable request approved by the institutional review boards.

## Supplementary information

**Additional file 1.** Primary parameters of the imaging sequences and the details of the feature information.

## Data Availability

The data sets used and/or analyzed during the current study are available from the corresponding author on reasonable request.

## Abbreviations

ADC: apparent diffusion coefficient
AUC: area under the curve
CEA: carcinoembryonic antigen
CM: co-occurrence matrices
CT: computed tomography
DWI: diffusion-weighted images
GLSZM: gray-level size zone matrix
LD: the longest diameter
LPD: the longest perpendicular diameter
LUAD: lung adenocarcinoma
LUSC: lung squamous cell carcinoma
MRI: magnetic resonance imaging
NGTDM: neighborhood gray-tone difference matrix
NSCLC: non-small-cell lung cancer
OR: odds ratio
RLM: run length matrix
ROC: receiver operating characteristic
ROI: region of interest
SVM: support vector machine
SVM-RFE: support vector machine-based recursive feature elimination
T2WI: T2-weighted images

## References

[1] Su R, Zhang J, Liu X, Wei L (2019). Identification of expression signatures for non-small-cell lung carcinoma subtype classification. Bioinformatics.

[2] Bashir U, Kawa B, Siddique M, Mak SM, Nair A, Mclean E, Bille A, Goh V, Cook G (2019). Non-invasive classifcation of non-small cell lung cancer: a comparison between random forest models utilising radiomic and semantic features. Br J Radiol.

[3] Bray F, Ferlay J, Soerjomataram I, Siegel RL, Torre LA, Jemal A (2018). Global cancer statistics 2018: GLOBOCAN estimates of incidence and mortality worldwide for 36 cancers in 185 countries. CA Cancer J Clin.

[4] Hoffman PC, Mauer AM, Vokes EE (2000). Lung cancer. Lancet.

[5] Herbst RS, Heymach JV, Lippman SM (2008). Lung cancer. N Engl J Med.

[6] Ma Y, Feng W, Wu Z, Liu M, Zhang F, Liang Z, Cui C, Huang J, Li X, Guo X (2018). Intra-tumoural heterogeneity characterization through texture and colour analysis for differentiation of non-small cell lung carcinoma subtypes. Phys Med Biol.

[7] Zhu X, Dong D, Chen Z, Fang M, Zhang L, Song J, Yu D, Zang Y, Liu Z, Shi J (2018). Radiomic signature as a diagnostic factor for histologic subtype classification of non-small cell lung cancer. Eur Radiol.

[8] Mahon RN, Hugo GD, Weiss E (2019). Repeatability of texture features derived from magnetic resonance and computed tomography imaging and use in predictive models for non-small cell lung cancer outcome. Phys Med Biol.

[9] Sun W, Jiang M, Dang J, Chang P, Yin FF (2018). Effect of machine learning methods on predicting NSCLC overall survival time based on Radiomics analysis. Radiat Oncol.

[10] Starkov P, Aguilera T, Golden D, Shultz D, Trakul N, Maxim P, Le Q-T, Loo BW, Diehn M, Depeursinge A, Rubin DL (2018). The use of texture-based radiomics CT analysis to predict outcomes in early-stage non-small cell lung cancer treated with stereotactic ablative radiotherapy. Br J Radiol.

[11] Sollini M, Cozzi L, Antunovic L, Chiti A, Kirienko M (2017). PET Radiomics in NSCLC: state of the art and a proposal for harmonization of methodology. Sci Rep.

[12] Shen C, Liu Z, Guan M, Song J, Lian Y, Wang S, Tang Z, Dong D, Kong L, Wang M (2017). 2D and 3D CT radiomics features prognostic performance comparison in non-small cell lung cancer. Transl Oncol.

[13] Liu LP, Zhang XX, Cui LB, Li J, Yang JL, Yang HN, Zhang Y, Zhou Y, Tang X, Qi S (2017). Preliminary comparison of diffusion-weighted MRI and PET/CT in predicting histological type and malignancy of lung cancer. Clin Resp J.

[14] Zhang J, Cui LB, Tang X, Ren XL, Shi JR, Yang HN, Zhang Y, Li ZK, Wu CG, Jian W (2014). DW MRI at 3.0 T versus FDG PET/CT for detection of malignant pulmonary tumors. Int J Cancer.

[15] Zhang X, Xu X, Tian Q, Li B, Wu Y, Yang Z, Liang Z, Liu Y, Cui G, Lu H (2017). Radiomics assessment of bladder cancer grade using texture features from diffusion-weighted imaging. J Magn Reson Imaging.

[16] Xu X, Zhang X, Tian Q, Zhang G, Liu Y, Cui G, Meng J, Wu Y, Liu T, Yang Z, Lu H (2017). Three-dimensional texture features from intensity and high-order derivative maps for the discrimination between bladder tumors and wall tissues via MRI. Int J Comput Assist Radiol Surg.

[17] Xu X, Liu Y, Zhang X, Tian Q, Wu Y, Zhang G, Meng J, Yang Z, Lu H (2017). Preoperative prediction of muscular invasiveness of bladder cancer with radiomic features on conventional MRI and its high-order derivative maps. Abdom Radiol.

[18] Xu X, Zhang X, Tian Q, Wang H, Cui LB, Li S, Tang X, Li B, Dolz J, Ayed IB (2019). Quantitative identification of nonmuscle-invasive and muscle-invasive bladder carcinomas: a multiparametric MRI radiomics analysis. J Magn Reson Imaging.

[19] Xu X, Wang H, Du P, Zhang F, Li S, Zhang Z, Yuan J, Liang Z, Zhang X, Guo Y (2019). A predictive nomogram for individualized recurrence stratification of bladder cancer using multiparametric MRI and clinical risk factors. J Magn Reson Imaging.

[20] Gillies RJ, Kinahan PE, Hricak H (2016). Radiomics: images are more than pictures, they are data. Radiology.

[21] Huang L, Kong Q, Liu Z, Wang J, Kang Z, Zhu Y (2017). The diagnostic value of MR imaging in differentiating T staging of bladder cancer: a meta-analysis. Radiology.

[22] Li Q, Bai H, Chen Y, Sun Q, Liu L, Zhou S, Wang G, Liang C, Li ZC (2017). A fully-automatic multiparametric radiomics model: towards reproducible and prognostic imaging signature for prediction of overall survival in glioblastoma multiforme. Sci Rep.

[23] Li H, Zhu Y, Burnside ES, Huang E, Drukker K, Hoadley KA, Fan C, Conzen SD, Zuley M, Net JM (2016). Quantitative MRI radiomics in the prediction of molecular classifications of breast cancer subtypes in the TCGA/TCIA data set. NPJ Breast Cancer.

[24] Ming F, Hui L, Shijian W, Bin Z, Juan Z, Lihua L, Alessandro W (2017). Radiomic analysis reveals DCE-MRI features for prediction of molecular subtypes of breast cancer. PLoS ONE.

[25] Zhuo E-H, Zhang W-J, Li H-J, Zhang G-Y, Jing B-Z, Zhou J, Cui C-Y, Chen M-Y, Sun Y, Liu L-Z (2019). Radiomics on multimodalities MR sequences can subtype patients with non-metastatic nasopharyngeal carcinoma (NPC) into distinct survival subgroups. Eur Radiol.

[26] Zhang X, Lu H, Tian Q, Feng N, Yin L, Xu X, Du P, Liu Y (2019). A radiomics nomogram based on multiparametric MRI might stratify glioblastoma patients according to survival. Eur Radiol.

[27] Haralick RM, Shanmugam K, Dinstein IH (1973). Textural features for image classification. IEEE Trans Syst Man Cybern.

[28] Galloway MM (1975). Texture analysis using gray level run lengths. Comput Graph Image Process.

[29] Amadasun M, King R (1989). Texural features corresponding to texural properties. IEEE Trans Syst Man Cybern.

[30] Thibault G, Angulo J, Meyer F (2014). Advanced statistical matrices for texture characterization: application to cell classification. IEEE Trans Biomed Eng.

[31] Wang H, Pui M, Guo Y, Yang D, Pan B, Zhou X (2014). Diffusion-weighted MRI in bladder carcinoma: the differentiation between tumor recurrence and benign changes after resection. Abdom Imaging.

[32] Rosenkrantz AB, Haghighi M, Horn J, Naik M, Hardie AD, Somberg MB, Melamed J, Xiao GQ, Huang WC, Taouli B (2013). Utility of quantitative MRI metrics for assessment of stage and grade of urothelial carcinoma of the bladder: preliminary results. AJR Am J Roentgenol.

[33] Wang H, Pui MH, Guan J, Li S, Lin J, Pan B, Guo Y (2016). Comparison of early submucosal enhancement and tumor stalk in staging bladder urothelial carcinoma. AJR Am J Roentgenol.

[34] Liu Y, Xu X, Yin L, Zhang X, Li L, Lu H (2017). Relationship between glioblastoma heterogeneity and survival time: an MR imaging texture analysis. Am J Neuroradiol.

[35] Chen YP, Chan ATC, Le QT, Blanchard P, Sun Y, Ma J (2019). Nasopharyngeal carcinoma. Lancet.

[36] Cui L-B, Liu L, Wang H-N, Wang L-X, Guo F, Xi Y-B, Liu T-T, Li C, Tian P, Liu K (2018). Disease definition for schizophrenia by functional connectivity using radiomics strategy. Schizophr Bull.

[37] Fehr D, Veeraraghavan H, Wibmer A, Gondo T, Matsumoto K, Vargas H, Sala E, Hricak H, Deasy J (2015). Automatic classification of prostate cancer Gleason scores from multiparametric magnetic resonance images. Proc Natl Acad Sci USA.

[38] Chatterjee A, He D, Fan X, Antic T, Jiang Y, Eggener S, Karczmar GS, Oto A (2019). Diagnosis of prostate cancer by use of MRI-derived quantitative risk maps: a feasibility study. Am J Roentgenol.

[39] Huang S, Cai N, Pacheco PP, Narrandes S, Wang Y, Xu W (2018). Applications of support vector machine (SVM) learning in cancer genomics. Cancer Genomics Proteomics.

[40] Burges CJC (1998). A tutorial on support vector machines for pattern recognition. Data Min Knowl Disc.

[41] Han H, Jiang X (2014). Overcome support vector machine diagnosis overfitting. Cancer Inform.

[42] Linning E, Lu L, Li L, Yang H, Schwartz LH, Zhao B (2019). Radiomics for classifying histological subtypes of lung cancer based on multiphasic contrast-enhanced computed tomography. J Comput Assist Tomogr.

[43] Lyssek-Boroń A, Wylęgała A, Polanowska K, Krysik K, Dobrowolski D (2017). Longitudinal changes in retinal nerve fiber layer thickness evaluated using avanti Rtvue-XR optical coherence tomography after 23G vitrectomy for epiretinal membrane in patients with open-angle glaucoma. J Healthcare Eng.

[44] Krysik K, Dobrowolski D, Polanowska K, Lyssek-Boroń A, Wylęgała EA (2017). Measurements of corneal thickness in eyes with pseudoexfoliation syndrome: comparative study of different image processing protocols. J Healthcare Eng.

[45] Zhang L, Wang J, Guo X, Qin B, Yi G, Liu Y, Fu S, Wang G (2018). Three-dimensional (3D) computed tomographic (CT) assessment of the sustentaculum tail to find distinctive characteristics: implications for surgery. Med Sci Monit.

[46] Krysik K, Dobrowolski D, Stanienda-Sokół K, Wylegala E, Boron A (2019). Scheimpflug camera and swept-source optical coherence tomography in pachymetry evaluation of diabetic patients. J Ophthalmol.

[47] Dolz J, Xu X, Rony J, Yuan J, Liu Y, Granger E, Desrosiers C, Zhang X, Ayed IB, Lu H (2018). Multiregion segmentation of bladder cancer structures in MRI with progressive dilated convolutional networks. Med Phys.

[48] Zheng J, Kong J, Wu S, Li Y, Cai J, Yu H, Xie W, Qin H, Wu Z, Huang J, Lin T (2019). Development of a noninvasive tool to preoperatively evaluate the muscular invasiveness of bladder cancer using a radiomics approach. Cancer.

[49] Wu S, Zheng J, Li Y, Yu H, Shi S, Xie W, Liu H, Su Y, Huang J, Lin T (2017). A radiomics nomogram for the preoperative prediction of lymph node metastasis in bladder cancer. Clin Cancer Res.

[50] Lambin P, Leijenaar RTH, Deist TM, Peerlings J, de Jong EEC, van Timmeren J, Sanduleanu S, Larue R, Even AJG, Jochems A (2017). Radiomics: the bridge between medical imaging and personalized medicine. Nat Rev Clin Oncol.

[51] Jiang Y, Yuan Q, Lv W, Xi S, Huang W, Sun Z, Chen H, Zhao L, Liu W, Hu Y (2018). Radiomic signature of (18)F fluorodeoxyglucose PET/CT for prediction of gastric cancer survival and chemotherapeutic benefits. Theranostics.

[52] Wu S, Zheng J, Li Y, Wu Z, Shi S, Huang M, Yu H, Dong W, Huang J, Lin T (2018). Development and validation of an MRI-based radiomics signature for the preoperative prediction of lymph node metastasis in bladder cancer. EBioMedicine.

[53] Gupta V, Mittal M (2019). R-Peak detection in ECG signal using Yule-Walker and principal component analysis. IETE J Res.

[54] Gupta V, Mittal M (2019). QRS complex detection using STFT, chaos analysis, and PCA in standard and real-time ECG databases. J Instit Eng B.

[55] Kora P, Krishna KSR. Myocardial infarction detection using magnitude squared coherence and Support Vector Machine. In: Medical Imaging, m-Health and Emerging Communication Systems (MedCom); 2014, p. 382–5. 10.1109/medcom.2014.7006037.

[56] Soukup V, Capoun O, Cohen D, Hernandez V, Burger M, Comperat E, Gontero P, Lam T, Mostafid AH, Palou J (2018). Risk stratification tools and prognostic models in non-muscle-invasive bladder cancer: a critical assessment from the European Association of Urology Non-muscle-invasive Bladder Cancer Guidelines Panel. Eur Urol Focus.

