# Supplementary material for: Elaboration of a multimodal MRI-based radiomics signature for the preoperative prediction of the histological subtype in patients with non-small-cell lung cancer
Source: Biomed Eng Online. 2020 Jan 21;19:5. doi: 10.1186/s12938-019-0744-0 (PMC6975040; doi:10.1186/s12938-019-0744-0)
Supplement: Supplementary file 1 — Additional file 1. Primary parameters of the imaging sequences and the details of the feature information. [file 12938_2019_744_MOESM1_ESM.docx]

**Primary Parameters of the Imaging Sequences and the Details of the Feature Information**

**I. Primary parameters of the multiparametric MRI performed**

All patients underwent MRI by using a 1.5 T scanner (MAGNETOM Aera, Siemens Medical Solutions, Erlangen) with an 8-channel phased-array torso coil. MRI sequences, including T2W and DW imaging sequences, were performed to obtain the corresponding images. The main parameters of the T2W sequence included: the sequence name “'t2_blade_fs_tra_p2_trig_320-LUNG'”, TR 2200 ms, TE 86 ms, Slice thickness 5 mm, Space between slices 1 mm, FOV 350 mm × 350 mm. The main parameters of the DW sequence included: the sequence name “ep2d_diff_stir_b50_800_p2_TRACEW_DFC”, b value 50 and 800 s/mm^2^, TR 6800 ms, TE 63 ms, Slice thickness 5 mm, Space between slices 0.25 mm, FOV 400 mm × 400 mm.

The corresponding ADC maps were calculated using a custom-developed MATLAB R2015b package to solve the following equation:

$S(b_{50})/S(b_{800})=\mathrm{e}^{(b_{800}\times ADC)}$ (1)

where S(b800) and S(b50) represent the signal intensity of a certain voxel in the presence and absence of diffusion sensitization, respectively.

**II. Feature Extraction**

After ROI delineation, the radiomics features, including the first-order features [[1-5](#_ENREF_1)] (histogram features), second-order features[[2-4](#_ENREF_2), [6](#_ENREF_6)] (Haralick features extracted from the co-occurrence matrix, CM features hereafter), and higher-order features [[2](#_ENREF_2), [4](#_ENREF_4), [7-11](#_ENREF_7)], including features extracted from the run-length matrix (RLM), neighborhood gray-tone difference matrix (NGTDM) and gray level size zone matrix (GLSZM), i.e., RLM features, NGTDM features and GLSZM features hereafter, were calculated from each ROI set to fully characterize the tissue distribution patterns within the tumor region. Specifically, prior to second-order and higher-order feature extraction, the image intensity was discretized and normalized to five standard grayscales (8, 16, 32, 64 and 128). Then, these four feature groups were extracted from the ROIs using each normalized grayscale.

Eight widely used histogram-based features [[1](#_ENREF_1), [2](#_ENREF_2), [4](#_ENREF_4)], including the mean, entropy, uniformity, standard deviation, smoothness, skewness, third-order moment and kurtosis, were applied to fully describe the global texture patterns within the tumor. These features are listed in Table S1.

***CM features***

CM features were proposed by Haralick et al.[[6](#_ENREF_6)]. They describe the pixel-paired distribution within images, which may reflect the local heterogeneity of tissues [[4](#_ENREF_4), [6](#_ENREF_6), [12](#_ENREF_12)]. For each ROI, four CMs were computed along four principal directions (0°, 45°, 90°, and 135°) with a distance between two pixels set as “1”. For each CM, Haralick et al. proposed 13 measures to characterize the tissue patterns [[1](#_ENREF_1), [6](#_ENREF_6), [12](#_ENREF_12)]. Therefore, for each Haralick measure, four values were eventually obtained from the four CMs. To keep the features rotationally invariant, for each measure, the average, range (difference between the maximum and minimum values) and standard deviation of its four values were calculated [[6](#_ENREF_6), [12](#_ENREF_12)]. Eventually, a total of 39 CM features were obtained (see Table S1).

***RLM features***

The run-length is used to evaluate the contiguous gray level along a predefined direction. Therefore, the RLM features are capable of describing the regional pixel distribution within the ROI and can thus reflect the regional heterogeneity of tissues.

For a given picture *Image I*, four RLMs can be computed along four principal directions (0°, 45°, 90°, and 135°). In each RLM, the matrix element *(m, n)* specifies the number of times that the picture contains a run of length *n* in a given direction, consisting of points having the gray-level *m*. Then, similar to the CM features, we calculated the average, range and standard deviation of the 13 measures of the four RLMs, constituting 33 RLM features in this group (Table S1).

***NGTDM features***

Textural features corresponding to the visual perception of image texture are highly desirable for pattern recognition and artificial intelligence[[10](#_ENREF_10)]. Neighborhood gray tone difference matrix (NGTDM)-based features were generated by Amadasun and King [[10](#_ENREF_10)], the purpose of which is to quantitatively describe the visual perception properties of image texture and to build a bridge between images and interpretation. The following five metrics are involved in this feature group:

(1) *NGTDM Coarseness*: Coarseness is the most fundamental property of texture, and in a narrow sense, it is used to imply texture [[10](#_ENREF_10)]. If the texture of an image is coarser, it means the primitives or basic patterns of the texture are large, which, in turn, tends to yield a texture with a high degree of local uniformity in intensity [[10](#_ENREF_10)].

(2) *NGTDM Contrast:* An image has a high level of contrast if areas of different gray levels are clearly visible [[10](#_ENREF_10)]. Thus, high contrast means that the intensity difference between neighboring regions is large [[10](#_ENREF_10)] and vice versa.

(3) *NGTDM Busyness:* A busy texture is one in which there are rapid changes in intensity from one pixel to neighboring pixels, which means that the spatial frequency of intensity changes in the region is very high [[10](#_ENREF_10)].

(4) *NGTDM Complexity*: Complexity indicates the visual information content of a texture. A texture is considered complex if the information content is high [[10](#_ENREF_10)].

(5) *NGTDM Strength:* A texture is generally referred to as strong when the primitives of the texture are easily definable and clearly visible [[10](#_ENREF_10)]. All these features are listed in Table S1.

***GLSZM features***

Gray-level size-zone matrix (GLSZM)-based features were generated and described by Thibault et al. [[4](#_ENREF_4), [8](#_ENREF_8), [11](#_ENREF_11)]. A GLSZM describes the number of homogeneous connected areas of a certain size and intensity within the region of interest (ROI) [[4](#_ENREF_4)]. The element *p(i, j)* of the GLSZM represents the number of connected regions of gray level *i* and size *j*. The features derived from the GLSZM, therefore, describe the homogeneous regions within the tumor ROI and can be used to quantitatively assess the tumor heterogeneity in an ROI [[4](#_ENREF_4)]. The feature group contains 15 widely reported metrics (Table S1).

**Table S1 Radiomic features extracted from each modality**

| Feature category | Feature ID | Description |
| --- | --- | --- |
| Histogram | H1 | Mean |
|  | H2 | Entropy |
|  | H3 | Uniformity |
|  | H4 | Standard deviation |
|  | H5 | Smoothness |
|  | H6 | Skewness |
|  | H7 | Third order moment |
|  | H8 | kurtosis |
| CM | CM1 | Energy |
|  | CM 2 | Contrast |
|  | CM 3 | Correlation |
|  | CM 4 | Variance |
|  | CM 5 | Inverse difference moment |
|  | CM 6 | Sum average |
|  | CM 7 | Sum variance |
|  | CM 8 | Sum entropy |
|  | CM 9 | Entropy |
|  | CM 10 | Difference variance |
|  | CM 11 | Difference entropy |
|  | CM 12 | Information measures I of correlation |
|  | CM 13 | Information measures II of correlation |
|  | CM 14～26 | Range of the corresponding features listed above |
|  | CM 27～39 | Standard deviation of the corresponding features listed above |
| RLM | RLM1 | Short run emphasis |
|  | RLM 2 | Long run emphasis |
|  | RLM 3 | Gray-level non-uniformity |
|  | RLM 4 | Run length non-uniformity |
|  | RLM 5 | Run percentage |
|  | RLM 6 | Low gray-level run emphasis |
|  | RLM 7 | High gray-level run emphasis |
|  | RLM 8 | Short run low gray-level emphasis |
|  | RLM 9 | Short run high gray-level emphasis |
|  | RLM 10 | Long run low gray-level emphasis |
|  | RLM 11 | Long run high gray-level emphasis |
|  | RLM 12～22 | Range of corresponding features listed above |
|  | RLM 23～33 | Standard deviation of corresponding features listed above |
| NGTDM | N1 | NGTDM Coarseness |
|  | N2 | NGTDM Contrast |
|  | N3 | NGTDM Busyness |
|  | N4 | NGTDM Complexity |
|  | N5 | NGTDM Strength |
| GLSZM | SZ1 | Small Area Emphasis (SAE) |
|  | SZ2 | Large Area Emphasis (LAE) |
|  | SZ3 | Low Intensity Emphasis (LIE) |
|  | SZ4 | High Intensity Emphasis (HIE) |
|  | SZ5 | Low Intensity Small Area Emphasis (LISAE) |
|  | SZ6 | High Intensity Small Area Emphasis (HISAE) |
|  | SZ7 | Low Intensity Large Area Emphasis (LILAE) |
|  | SZ8 | High Intensity Large Area Emphasis (HILAE) |
|  | SZ9 | Intensity Nonuniformity (IN) |
|  | SZ10 | Intensity Nonuniformity Normalized (INN) |
|  | SZ11 | Size Zone Nonuniformity (SZN) |
|  | SZ12 | Size Zone Percentage (SZP) |
|  | SZ13 | Intensity Variance (IV) |
|  | SZ14 | Size Zone Variance (SZV) |
|  | SZ15 | Size Zone Entropy (SZE) |

Prior to CM, RLM, NGTDM and GLSZM feature extraction, the grayscale normalization process is required. In this study, five commonly used grayscales, i.e., 8, 16, 32, 64, and 128, were adopted to fully characterize the tissue distribution patterns with the tumor ROI. Therefore, for each image group (for instance, the T2-weighted MR image group), 468 features were computed, including 8 histogram features, 195 CM features (39×5), 165 RLM features (33×5), 25 RLM features (5×5), and 75 RLM features (15×5). A total of 1404 features (468×3) were finally calculated (Table S1). We allocated a specific feature ID to each feature. If a feature was extracted from T2W images, the name of this feature became “T2W-featureID-GL”, where GL stands for grayscale. For example, the feature “DW-RLM32-16GL” denotes the standard deviation of “Long run low gray-level emphasis” in the RLM feature category calculated from the 16 grayscale normalized DW images.

**III. References**

1. Xu X, Liu Y, Zhang X, Tian Q, Wu Y, Zhang G, et al. Preoperative prediction of muscular invasiveness of bladder cancer with radiomic features on conventional MRI and its high-order derivative maps. Abdominal radiology. 2017; 42: 1896-905.

2. Lambin P, Rios-Velazquez E, Leijenaar R, Carvalho S, van Stiphout RGPM, Granton P, et al. Radiomics: Extracting more information from medical images using advanced feature analysis. European journal of cancer (Oxford, England : 1990). 2012; 48: 441-6.

3. Zhang X, Xu X, Tian Q, Li B, Wu Y, Yang Z, et al. Radiomics assessment of bladder cancer grade using texture features from diffusion-weighted imaging. Journal of magnetic resonance imaging : JMRI. 2017; 46: 1281-8.

4. Lambin P, Leijenaar RTH, Deist TM, Peerlings J, de Jong EEC, van Timmeren J, et al. Radiomics: the bridge between medical imaging and personalized medicine. Nature reviews Clinical oncology. 2017; 14: 749-62.

5. Xu X, Wang H, Du P, Zhang F, Li S, Zhang Z, et al. A predictive nomogram for individualized recurrence stratification of bladder cancer using multiparametric MRI and clinical risk factors. Journal of Magnetic Resonance Imaging. 2019 Apr 13; 0.

6. Haralick RM, Shanmugam K, Dinstein IH. Textural Features for Image Classification. IEEE Transactions on Systems, Man, and Cybernetics. 1973; SMC-3: 610-21.

7. Galloway MM. Texture Analysis Using Gray Level Run Lengths. Computer Graphics and Image Processing. 1975; 4: 172-9

8. Thibault G, Angulo J, Meyer F. Advanced statistical matrices for texture characterization: Application to DNA chromatin and microtubule network classification. IEEE International Conference on Image Processing; 2011. p. 53-6.

9. Thibault G, Angulo J, Meyer F. Advanced statistical matrices for texture characterization: application to cell classification. IEEE Transactions on Biomedical Engineering. 2014; 61: 630-7.

10. Amadasun M, King R. Texural Features Corresponding to Texural Properties. IEEE Transactions on Systems, Man, and Cybernetics. 1989; 19: 1264-74.

11. Thibault G, Fertil B, Navarro C, Pereira S, Levy N, Sequeira J, et al. Texture Indexes and Gray Level Size Zone Matrix Application to Cell Nuclei Classification. In Pattern Recognition and Information Processing (PRIP. 2009.

12. Xu X, Zhang X, Tian Q, Wang H, Cui L-B, Li S, et al. Quantitative Identification of Nonmuscle-Invasive and Muscle-Invasive Bladder Carcinomas: A Multiparametric MRI Radiomics Analysis. Journal of magnetic resonance imaging : JMRI. 2018; 10.1002/jmri.26327.
